# Co-fermentation using Recombinant *Saccharomyces cerevisiae* Yeast Strains Hyper-secreting Different Cellulases for the Production of Cellulosic Bioethanol

**DOI:** 10.1038/s41598-017-04815-1

**Published:** 2017-06-30

**Authors:** Cho-Ryong Lee, Bong Hyun Sung, Kwang-Mook Lim, Mi-Jin Kim, Min Jeong Sohn, Jung-Hoon Bae, Jung-Hoon Sohn

**Affiliations:** 1 Cell Factory Research Center, Korea Research Institute of Bioscience and Biotechnology (KRIBB), Daejeon, 34141 Republic of Korea; 2 Department of Biosystems and Bioengineering, KRIBB School of Biotechnology, Korea University of Science and Technology (UST), Daejeon, 34113 Republic of Korea

## Abstract

To realize the economical production of ethanol and other bio-based chemicals from lignocellulosic biomass by consolidated bioprocessing (CBP), various cellulases from different sources were tested to improve the level of cellulase secretion in the yeast *Saccharomyces cerevisiae* by screening an optimal translational fusion partner (TFP) as both a secretion signal and fusion partner. Among them, four indispensable cellulases for cellulose hydrolysis, including *Chaetomium thermophilum* cellobiohydrolase (CtCBH1), *Chrysosporium lucknowense* cellobiohydrolase (ClCBH2), *Trichoderma reesei* endoglucanase (TrEGL2), and *Saccharomycopsis fibuligera* β-glucosidase (SfBGL1), were identified to be highly secreted in active form in yeast. Despite variability in the enzyme levels produced, each recombinant yeast could secrete approximately 0.6–2.0 g/L of cellulases into the fermentation broth. The synergistic effect of the mixed culture of the four strains expressing the essential cellulases with the insoluble substrate Avicel and several types of cellulosic biomass was demonstrated to be effective. Co-fermentation of these yeast strains produced approximately 14 g/L ethanol from the pre-treated rice straw containing 35 g/L glucan with 3-fold higher productivity than that of wild type yeast using a reduced amount of commercial cellulases. This process will contribute to the cost-effective production of bioenergy such as bioethanol and biochemicals from cellulosic biomass.

## Introduction

Production of biofuels such as bioethanol from lignocellulosic biomass is important for the effective reuse of natural resources and expansion of energy sources; however, from an economic perspective, its popularization is largely dependent on the ability to compete with the price of petroleum^1^. The bioethanol production process with yeast involves pre-treatment of the lignocellulose, enzymatic hydrolysis of the biomass into reducing sugars, and ethanol fermentation using hexose or pentose, which are sugar monomers in the biomass^2^. To reduce the cost of ethanol production, simplified processes such as simultaneous saccharification and fermentation (SSF), simultaneous saccharification and co-fermentation (SSCF), and consolidated bioprocessing (CBP) have been proposed as alternatives to separate the hydrolysis and fermentation steps^3^. In SSF, the continuous consumption of sugars produced from lignocellulose by enzymatic hydrolysis and the consequent production of ethanol can prevent the feedback inhibition of the cellulolytic enzymes and decrease the contamination^4, 5^. The SSCF method combines SSF and the cooperative fermentation of pentose as well as hexose. An ethanol yield of over 70% was achieved by SSCF using a xylose-fermenting *Saccharomyces cerevisiae* strain with commercial cellulases^6^.

One of the main challenges facing industrial bioethanol production from lignocellulose is the large amount of cellulase enzymes required for the hydrolysis of cellulose^4, 7^. The cost of cellulase alone is estimated to be as high as 25–50% of the total ethanol production costs^8^. As a strategy for the dramatic reduction of the enzyme cost associated with cellulosic bioethanol production, a technical consolidation of cellulase production, saccharification, and fermentation using an engineered microorganism in a single reactor has been proposed^9^. CBP developments are classified into two categories: (i) ethanol production by naturally cellulolytic organisms such as *Trichoderma reesei*, *Clostridium* sp., and *Bacillus subtilis*, and (ii) cellulase production by naturally fermentative organisms such as *S. cerevisiae*, *Pichia stipitis*, and *Kluyveromyces marxianus* ^10^. Because of the difficulty of introducing the ethanol fermentation pathway into cellulolytic organisms, most of the CBP developments proposed thus far have focused on the latter category^9^. In general, three kinds of enzymes are needed to degrade cellulose: endoglucanase (EC 3.2.1.4; EGL), β-glucosidase (EC 3.2.1.21; BGL), and cellobiohydrolase (EC 3.2.1.91; CBH)^11^. There are two major CBH classes: those of the glycosyl hydrolase families GH7 (also called CBH1) and GH6 (CBH2)^12^. Both classes of CBHs are used together because they synergistically act in cellulose hydrolysis^13, 14^.

There are three main types of strategies proposed to obtain cellulolytic ability by introducing cellulase genes into ethanologen microbes, such as mini-cellulosome, cell-surface display, and free-enzyme secretion^10, 15^. Fan *et al*.^16^ accomplished bioethanol production through the use of mini-cellulosomes, including EGL, CBHs, and BGL, on the cell surface in *S. cerevisiae*, whereas a low ethanol production yield (about 27%) was obtained from Avicel, with similar crystallinity of pre-treated cellulosic biomass^17^. In another attempt, Liu *et al*.^18^ developed a cell-surface display system containing four different cellulases (EGL, BGL, CBH1, and CBH2) in *S. cerevisiae*. Over 80% of the ethanol production yield from rice straw was achieved using cellulases displaying cells with the addition of the commercial cellulase. The average ethanol produced from Avicel by free cellulases was slightly higher than that accumulated by the cell-surface display system^18^. These two cellulase immobilization systems on the cell surface may cause inefficiency of processive enzymes, resulting in a decrease of the crystalline cellulose conversion rate compared to that of free enzymes systems, although these systems have certain benefits such as enzyme recycling and the close proximity of catalytic products^15^. In contrast, the free cellulase secretion strategy offers a method to avoid an argument about the physical restrictions associated with cell-surface display. Cellulolytic yeasts were constructed by the secretion of individual cellulases into the medium^19–21^; however, complete conversion of the insoluble cellulosic substrate by recombinant yeasts has not yet been achieved^15^. The key element to determining the performance of this system is the amount of enzymes secreted, because enzymes might be diffused away in a reactor^15^. Namely, efficient cellulase secretion is a prerequisite for industrial bioethanol or biochemical production from cellulosic biomass via yeast CBP.

To secrete heterologous cellulases in yeast, a native signal peptide or the signal peptide of *S. cerevisiae* mating factor alpha (MFα), Suc2, Pho5, and Inu of *K. marxianus* have been generally used^12, 22–25^. However, the secretion levels of each cellulase using the well-known secretion leaders in yeast are often not sufficient for CBP, thus making it impossible to displace commercial fungal cellulase required. The secretion of cellulases is highly dependent on the amino acid sequence or structure of the protein. The proper combination of a secretion leader and a target protein is required to maximize the final secretion level of a target protein. Recently, a novel protein secretion system was developed for the poorly secreted proteins in yeast by providing a protein-specific translational fusion partners (TFP) selected from yeast genome-wide secretion leader library^26^. This technology could improve the secretion level of many proteins and provide a broadened repertoire of unexplored secretion leaders from the yeast *S. cerevisiae*.

**Figure 1:**
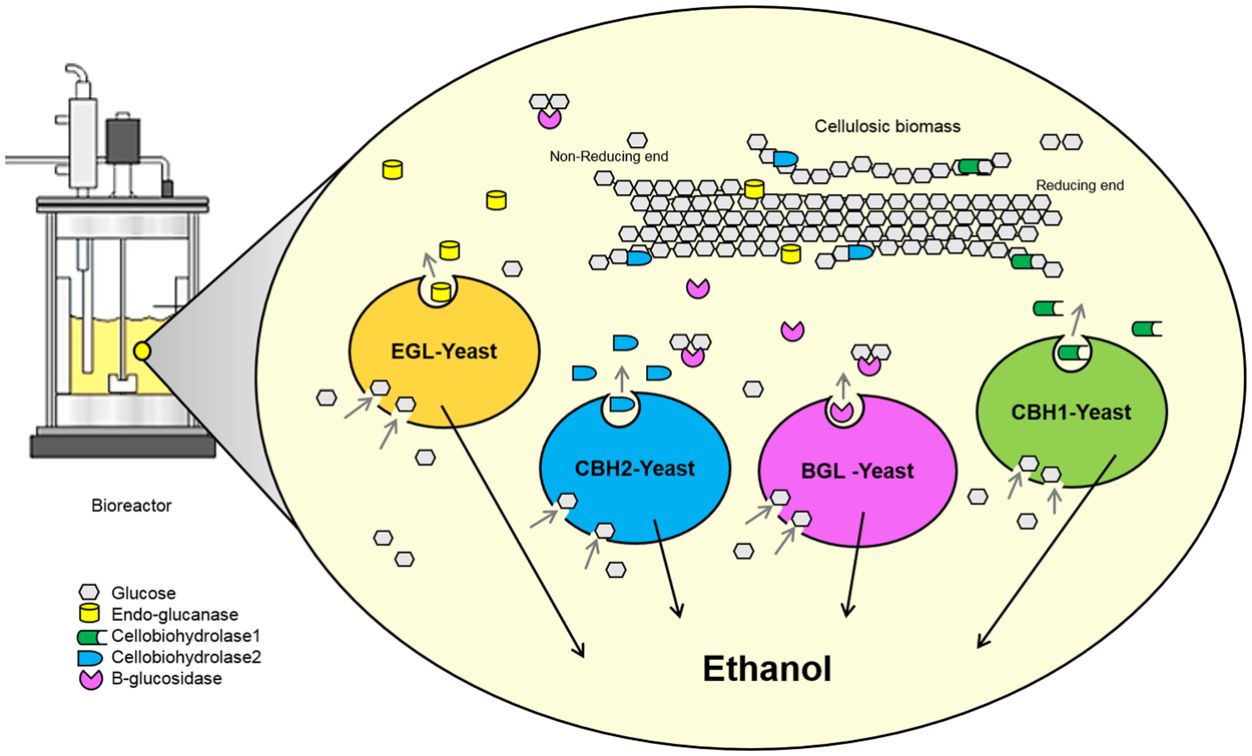
Schematic diagram of CBP by one-pot co-fermentation of recombinant yeasts. Recombinant yeasts secreting essential component of cellulases were co-fermented with cellulosic biomass in one bioreactor simultaneously. Glucose generated by the synergistic action of cellulases was used by each recombinant yeast as a carbon source for ethanol fermentation.

In this study, we successfully secreted four indispensable components of cellulase, including two CBHs, an EGL, and a BGL, by the selection of optimal TFPs from the TFP library, and analysed the cellulolytic activity of the secreted enzymes. Because the engineering of a single yeast to produce all cellulases like a fungal system has some challenges such as difficulty to construction, genetic instability, and low cellulase production, co-fermentation of yeasts secreting each cellulase was perfomed to provide all cellulase components for saccharification of biomass. The combination of the four yeast strains secreting the essential cellulases was applied to produce ethanol by CBP as shown in Fig. 1. This cellulase secretion system could reduce the total production cost for the cellulosic bioethanol and be useful for yeast biorefineries to produce valuable biochemicals from lignocellulosic biomass.

## Results

### TFP selection for the secretory expression of cellulases in *S. cerevisiae*

**Figure 2:**
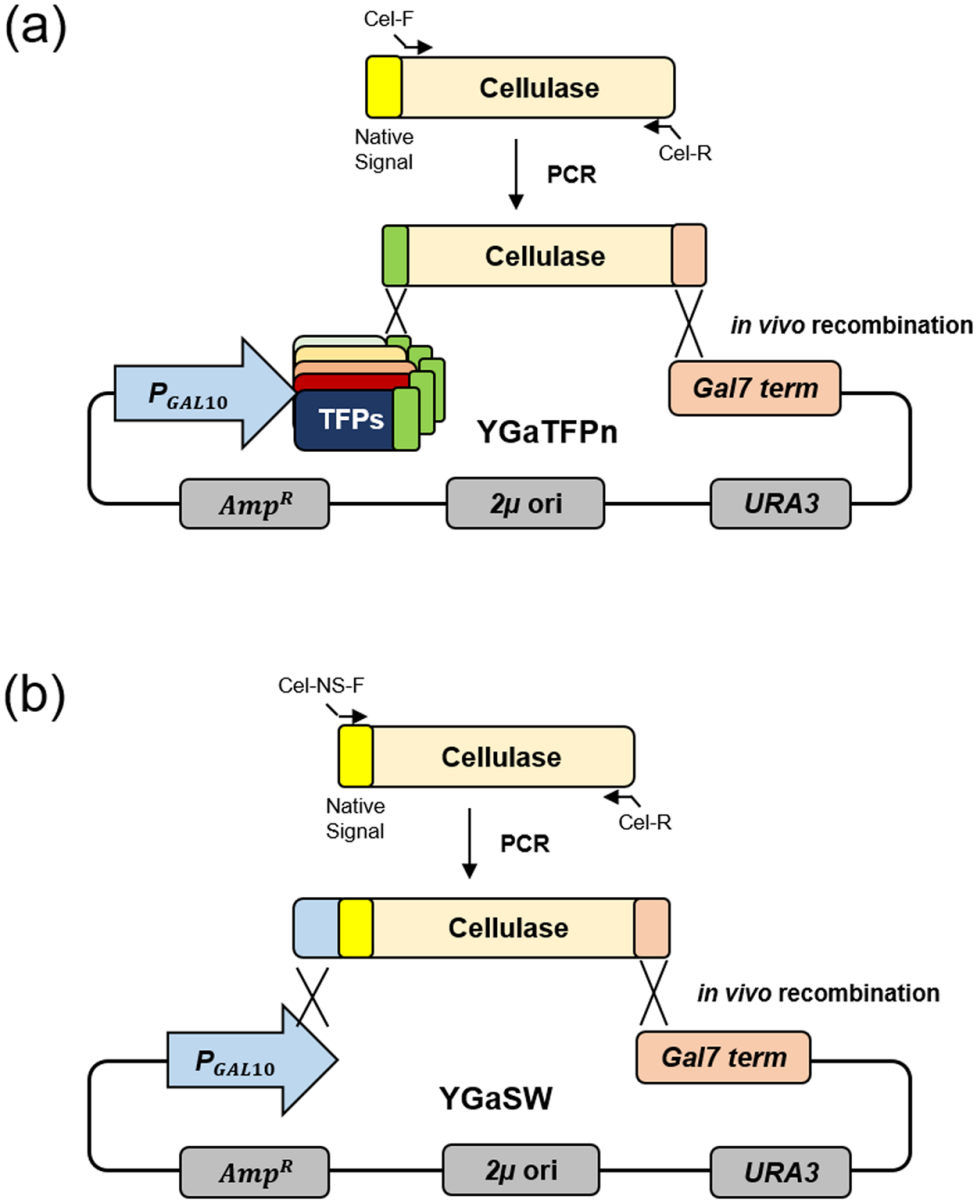
Diagram for vector construction by *in vivo* recombination for screening an optimal secretion signal. (**a**) The cellulase genes were flanked with the linker and terminator fragment by PCR with the primers Cel-F/Cel-R and transformed with the 24 *Swa*I-linearized TFP vectors. (**b**) For secretion of the cellulases with their native signal sequences, amplified cellulase genes containing the signal sequence were integrated into the *Swa*I-digested YGaSW vector by *in vivo* recombination.

**Figure 3:**
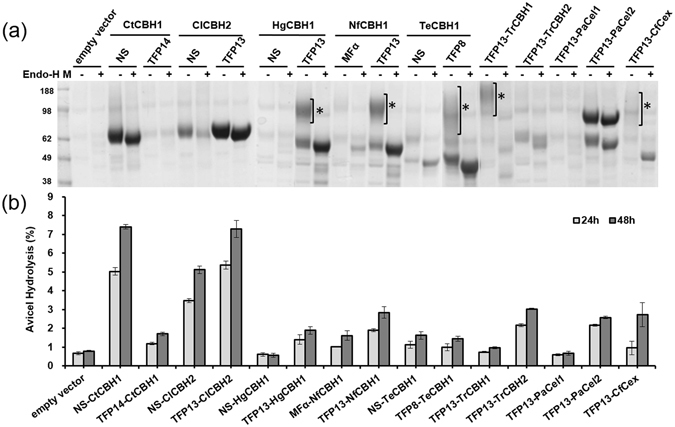
Secretion of exo-cellulases in *S. cerevisiae*. (**a**) Secretion of CBHs was analysed by SDS-PAGE using 100 μL of the test tube culture supernatants after freeze-drying. All samples were treated with (+) or without (−) endo-H for deglycosylation. The asterisks indicate the hyperglycosylated cellulases. (**b**) The degree of Avicel hydrolysis was detected based on the amount of glucose released by secreted CBHs with 0.5% (v/v) Novozyme A188 in 24 h (grey bars) and 48 h (black bars). Activity was expressed as the percentage of Avicel hydrolysed, as described previously^12^. Experiments were performed in triplicate and repeated three times.

**Figure 4:**
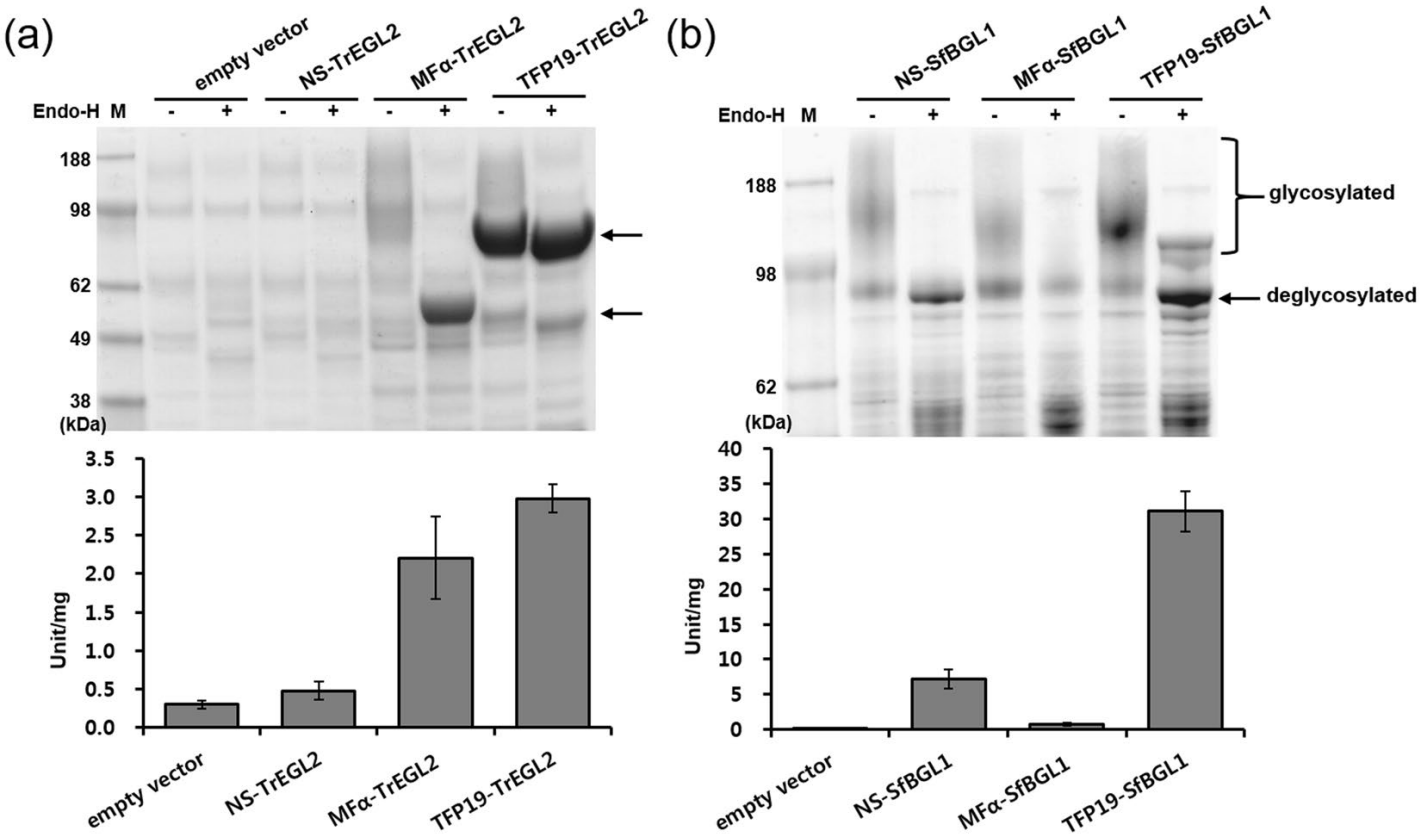
Secretion of TrEGL2 and SfBGL1 in *S. cerevisiae*. (**a**) Secretion of TrEGL2s was analysed by SDS-PAGE using 100 μL of the test tube culture supernatants after freeze-drying. The activity of CMC hydrolysis was detected based on the amount of reducing sugar released by secreted EGL. (**b**) Secretion levels of SfBGL1s were compared by SDS-PAGE. BGL activity for *p*-NPG hydrolysis was detected based on the amount of *p*-NP released by BGL. All samples were treated with (+) or without (−) endo-H for deglycosylation. Experiments were performed in triplicate and repeated three times.

For construction of recombinant *S. cerevisiae* strains that could secrete cellulases efficiently into the culture supernatant, we explored cellulase genes from various organisms, including 7 cellobiohydrolase 1 (CBH1)-encoding genes (Cel7A; PaCel1, HgCBH1, TeCBH1, NfCBH1, CtCBH1, TrCBH1, CfCex), 3 cellobiohydrolase 2 (CBH2)-encoding genes (Cel6A; PaCel2, ClCBH2, TrCBH2), and genes encoding an endoglucanase (TrEGL2) and a β-glucosidase (SfBGL1), respectively (Table 1), using the TFP system^26^. Each gene encoding the mature part of the cellulase was amplified using polymerase chain reaction (PCR) using the primers indicated in Supplementary Table S1, and cloned into the YGaTFPn vectors harbouring 24 different TFPs (Table 2) under the control of the *GAL10* promoter and *GAL7* terminator on a URA3-selectable episomal multi-copy vector, by *in vivo* recombination (Fig. 2a). The TFP vectors were designed to include the Kex2p processing site in the junction between the TFP and target cellulase to secrete the correctly processed mature cellulase. To compare the secretion efficiencies between the native secretion signal of cellulase and the TFPs, each cellulase gene with a native secretion signal was also amplified using PCR and cloned into the YGaSW vector that lacked a TFP but had the same backbone (Fig. 2b). After cultivation of each recombinant strain, the culture supernatants were analysed by sodium dodecyl sulphate-polyacrylamide gel electrophoresis (SDS-PAGE) and a cellulase activity test to compare the secretion levels and activities of the target cellulases secreted by the specific TFPs. Most of the TFPs could secrete correctly processed cellulases through Kex2p processing, but with different secretion levels (as one example, the secretion analysis of ClCBH2 is shown in Supplementary Fig. S1). The following transformants were identified as showing the best secretion of cellulases: TFP 13 for ClCBH2, HgCBH1, NfCBH1, CfCex, TrCBH1, TrCBH2, PaCel1, and PaCel2; TFP 8 for TeCBH1; the native signal sequence for CtCBH1; and TFP19 for TrEGL2 and SfBGL1 (Figs 3a and 4). Treatment with endoglycosidase H (endo-H) showed that several cellulases, including HgCBH1, NfCBH1, TeCBH1, TrCBH1, and CfCex were highly glycosylated by the yeast glycosylation system (Fig. 3a).

**Table 1:** Genes encoding the cellulases used in this study.

Gene	Source	Family	Function	Features	GenBank accession no.
TrCBH1	*Trichoderma reesei cbh1*	GH7	Cellobiohydrolase I	Signal peptide of 17 aa, CBM1 in C-term	[SwissProt:P62694]
HgCBH1	*Humicola grisea cbh1*	GH7	Cellobiohydrolase I	Signal peptide of 18 aa, CBM1 in C-term	[GenBank:CAA35159]
TeCBH1	*Talaromyces emersonii cbh1*	GH7	Cellobiohydrolase I	Signal peptide of 18 aa, No CBM1	[GenBank:AAL89553]
NfCBH1	*Neosartorya fischeri cbh1*	GH7	Cellobiohydrolase I	Signal peptide of 26 aa, CBM1 in C-term	[GenBank:XP_001258278]
CtCBH1	*Chaetomium thermophilum cbh1*	GH7	Cellobiohydrolase I	Signal peptide of 18 aa, CBM1 in C-term	[GenBank:CAM98448.1]
PaCel1	*Polyporus arcularius cbh1*	GH7	Cellobiohydrolase I	Signal peptide of 18 aa, No CBM1	[GenBank: BAF80326.1]
CfCex	*Cellulomonas fimi cbh*	GH10	Exo-beta-1,4-glucanase and Beta-1,4-xylanase	Signal peptide of 42 aa, CBM2 in N-term	[GenBank: AAA56792.1]
TrCBH2	*Trichoderma reesei cbh2*	GH6	Cellobiohydrolase II	Signal peptide of 24 aa, CBM1 in N-term	[SwissProt:P07987]
ClCBH2	*Chrysosporium lucknowense cbh2b*	GH6	Cellobiohydrolase II	Signal peptide of 17 aa, CBM1 in N-term	[GenBank:HH793136.1]
PaCel2	*Polyporus arcularius cbh2*	GH6	Cellobiohydrolase II	Signal peptide of 20 aa, CBM1 in N-term	[GenBank: BAF80327.1]
TrEGL2	*Trichoderma reesei egl2/cel5a*	GH5	Endo-1,4-glucanase	Signal peptide of 21 aa, CBM1 in N-term	[SwissProt:P07982]
SfBGL1	*Saccharomycopsis fibuligera bgl1*	GH3	Beta-glucosidase	Signal peptide of 17 aa, Fn3-like domain in C-term	[GenBank:ACH90244.1]

**Table 2:** List of translational fusion partners (TFPs) in 24 mini TFP library vectors.

TFP number	Gene name	Length^a^	Signal peptide length^a^	Characteristics^b^
1	YAR066	118	23	Pre-SS, N-gly, Ser, Ala-rich, GPI
2	YFR026c	130	18	Pre-SS, N-gly, TMD
3	CIS3	117	21	Pre-pro-SS, O-gly, PIR
4	DAN2	66	20	Pre-SS, CWP
5	SCW4	97	19	Pre-SS, CWP
6	MFα	93	19	Pre-pro SS
7	YGR106C	226	24	Pre-SS, N-gly, TMD
8	SRL1	64	19	Pre-SS, N-gly, O-gly, Ser, Thr-rich
9	SIM1-1	138	19	Pre-SS, N-gly, O-gly, Ser, Ala-rich, SUN family
10	OST3	199	22	Pre-SS, O-gly
11	Ynl190w	77	20	Pre-SS, N-gly, internal repeats, CWP
12	EMP24	94	19	Pre-SS, TMD
13	HSP150	174	18	Pre-pro SS
14	ECM33	68	19	Pre-SS, GPI
15	ATG27	157	19	Pre-SS, TMD
16	UTH1	98	17	Pre-SS, SUN family, Ser-rich
17	SED1	195	18	Pre-SS, GPI
18	BGL2	91	23	Pre-SS
19	SCW4	124	19	Pre-SS, CWP
20	CCW12	138	18	Pre-SS, CWP
21	FIT3	176	18	Pre-SS, GPI
22	YGP1	138	19	Pre-SS, N-gly, CWP
23	CCW14	115	22	Pre-SS, CWP
24	SED1	170	18	Pre-SS, GPI

^a^Number of amino acids.

^b^Pre-SS, pre secretion signal; Pre-pro-SS, pre-pro secretion signal; N-gly, N-glycosylation site; O-gly, o-glycosylation site; GPI, glycosyl phosphatidyl inositol anchor protein; PIR, protein internal repeats; CWP, cell wall protein; TMD, transmembrane domain.

### Activity of the secreted cellulases

To select strains producing each cellulase, the activity of the secreted CBHs was compared by measuring the quantity of glucose released from the crystalline cellulose Avicel PH-101 in the presence of a β-glucosidase, Novozyme A188. Avicel hydrolysis activities correlated with the protein secretion levels determined by SDS-PAGE, with some exceptional cases. Despite the greatly improved secretion levels of HgCBH1, NfCBH1, and TeCBH1 by TFPs as compared to those of the native signals or MFα, they showed relatively low Avicel-hydrolysing activity, which may be caused by hypergycosylation of heterologous proteins in yeast *S. cerevisiae*. Among the CBHs tested, CtCBH1 and ClCBH2 showed higher activities hydrolysing approximately 7.4% and 7.3% of 20 g/L Avicel, respectively (Fig. 3b). The Avicel hydrolysis activity of CtCBH1 secreted by the native signal sequence (NS-CtCBH1) was higher 4.3-fold compared to that of CtCBH1 secreted by TFP 14, which showed the highest activity among the CtCBH1s secreted by the TFPs. Protein secretion and the enzymatic hydrolysis activity of ClCBH2 by TFP 13 were 2.4-fold and 1.4-fold more efficient than those obtained by the native signal peptide and MFα, respectively. The cellulose hydrolysis activity of ClCBH2 secreted by TFP 13 (TFP13-ClCBH2) was approximately 2.4 and 2.8 times higher than that of TrCBH2 secreted by TFP 13 and of PaCel2 secreted by TFP 13, respectively. These results suggest that a native secretion signal of CtCBH1 is better than any other tested TFPs, while that of ClCBH2 is required to be replaced by TFP for efficient expression in yeast. Evaluation of the activity of secreted enzymes during cultivation showed that ClCBH2 and CtCBH1 reached their maximum activities after 36 h of growth on YPD medium (Supplementary Fig. S2). Therefore, all strains were cultured for two days before analysis. CBH1 and CBH2 have been reported to act synergistically in the hydrolysis of crystalline cellulose given that they hydrolyse the cellulose chain from different ends^12, 13^. Accordingly, we chose CtCBH1 and ClCBH2 as potentially useful CBHs for cellulase combinations in yeast to enhance cellulose hydrolysis. Using the selected yeast strains Y2805Δgal80/NS-CtCBH1 and Y2805Δgal80/TFP13-ClCBH2, approximately 22.1 mg/g dry cell weight (DCW) of CtCBH1 and 39.4 mg/g DCW of ClCBH2 could be produced from the fed-batch fermentations, respectively (Supplementary Fig. S3a and b).

In addition to the CBHs, we compared the amounts of BGL and EGL secreted into the culture supernatant by the signal peptide MFα, the native signal sequence, and TFPs for SfBGL1^21^ and TrEGL2^27, 28^. For EGL activity analysis, the secretion of EGL by the TFP system was analysed by incubating the cell-free culture supernatants of *S. cerevisiae* harbouring YGaTFPn-EGL with carboxymethylcellulose (CMC) and determining the amount of the reducing sugars formed. Among them, the yeast strain Y2805Δgal80, harbouring YGa-TFP19-TrEGL2, showed the highest EGL activity in the reaction using CMC. More specifically, Y2805Δgal80/TFP19-TrEGL2 showed 6.2-fold and 1.3-fold higher activity than that of YGa-NS-TrEGL2 and YGa-MFα-TrEGL2, respectively (Fig. 4a). Since the expected protein size of native TrEGL2 is around 44.2 kDa, MFα-TrEGL2 and TFP19-TrEGL2 were both secreted as fusion forms (55 kDa and 80 kDa) without Kex2p processing but nevertheless maintained their activities. Interestingly, TFP19-TrEGL2 was much less glycosylated than MFα-TrEGL2 as detected by treatment of endo-H. For BGL, the secreted enzyme activity was measured by incubation in cell-free yeast culture supernatants with cellobiose, and the amount of the reducing sugars formed was determined. The enzyme activity of SfBGL1 secreted by TFP 19 was higher 4.3-fold and 39.9-fold compared with that secreted by the native signal peptide and MFα, respectively (Fig. 4b). Most of the SfBGL1 secreted in yeast was highly glycosylated, but the glycosylation did not affect the activity. Fed-batch fermentations of Y2805Δgal80/TFP19-TrEGL2 and Y2805Δgal80/TFP19-SfBGL1 resulted in 1.2 g/L of TrEGL2 and 0.6 g/L of SfBGL1 from cell amounts of 40.5 and 43.5 g DCW in the fed-batch fermentations, respectively (Supplementary Fig. S3c and d).

Although previous studies have reported the use of a native signal sequence or heterologous signal peptide such as MFα^12^ for the secretion of cellulases, several TFPs specific to the target proteins were identified for the improvement of cellulase secretion, as shown in Figs 3 and 4. Based on the aforementioned results, we selected the yeast strains secreting NS-CtCBH1, TFP13-ClCBH2, TFP19-TrEGL2, and TFP19-SfBGL1 to prepare a cellulase cocktail for the hydrolysis of pre-treated lignocellulosic biomass, which was designated as KRIBB cellulase cocktail (KCC). The KCC1111 was constructed by mixing four types of cellulases in the same ratio (1:1:1:1).

### Saccharification of various types of crystalline biomass using the KCC

**Figure 5:**
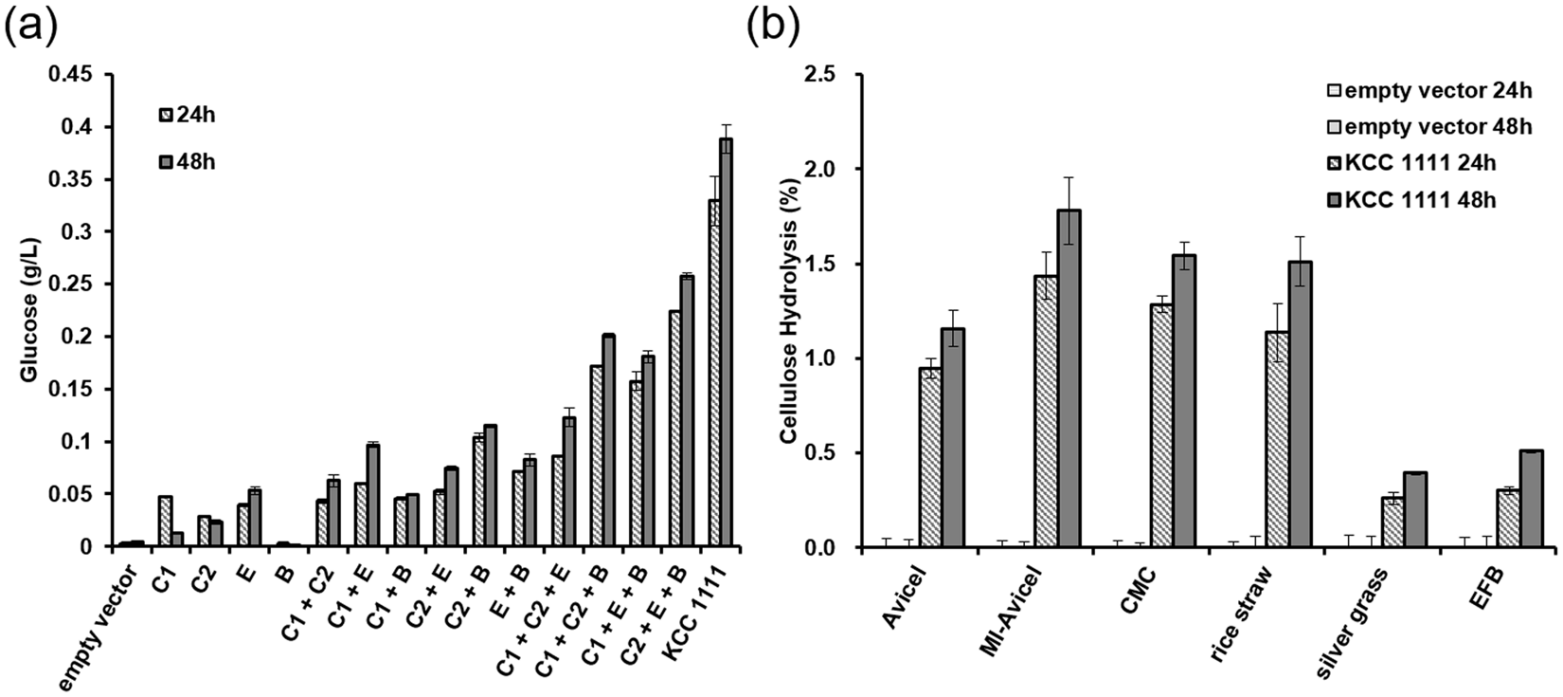
Cellulose hydrolysis of a recombinant cellulase cocktail. (**a**) The crystalline cellulose-hydrolysing activity of various cocktails was compared using 2% (w/v) Avicel. C1, C2, E, B, and KCC1111 represent CtCBH1, ClCBH2, TrEGL2, SfBGL1, and the KRIBB cellulase cocktail, respectively. (**b**) Cellulose hydrolysis activity of the cellulase cocktail was analysed by decomposing the cellulose into glucose from 2% (w/v) artificial substrates, including CMC and Avicel, and pre-treated biomass, including MI-Avicel, rice-straw, silver grass, and EFB. Experiments were performed in triplicate and repeated three times.

For verification of the synergistic effect of cellulases, the Avicel-hydrolysing activity of each cellulase or various cocktails containing two, three, or four ingredients was compared (Fig. 5a). Although the individual cellulases showed low Avicel-hydrolysing activity, it was increased in cocktails containing more than two ingredients. The KCC including all cellulases showed approximately 10-fold higher activity compared to those of single cellulases. This indicates that the recombinant cellulases produced in yeast work normally in the same manner as fungal cellulase, and the four cellulases are essential components for obtaining the full activity of the KCC. The accumulation of glucose from the KCC was evaluated following incubation with 2% of Avicel, 1-methylimidazole-treated Avicel (MI-Avicel), CMC, pre-treated rice straw, silver glass, or empty fruit bunch (EFB) of palm for 24 h and 48 h; the percent substrate hydrolysis after 48 h was 1.2%, 1.8%, 1.5%, 1.5%, 0.4%, and 0.5%, respectively (Fig. 5b). Hydrolysis of Avicel by the KCC was 25% of that resulting from the hydrolysis activity of CBH alone, probably owing to the lower activity of SfBGL1 than that of the commercial BGL used in Fig. 3b. For hydrolysis of the natural cellulosic biomass, the KCC showed 3-fold higher performance to pre-treated rice straw than the other two biomasses, silver grass, and EFB. The different efficiencies of hydrolysis by the KCC may be caused by the different compositions of biomass and efficiency of the pre-treatment.

### Ethanol production properties of the cellulase-secreting yeast mixture

**Figure 6:**
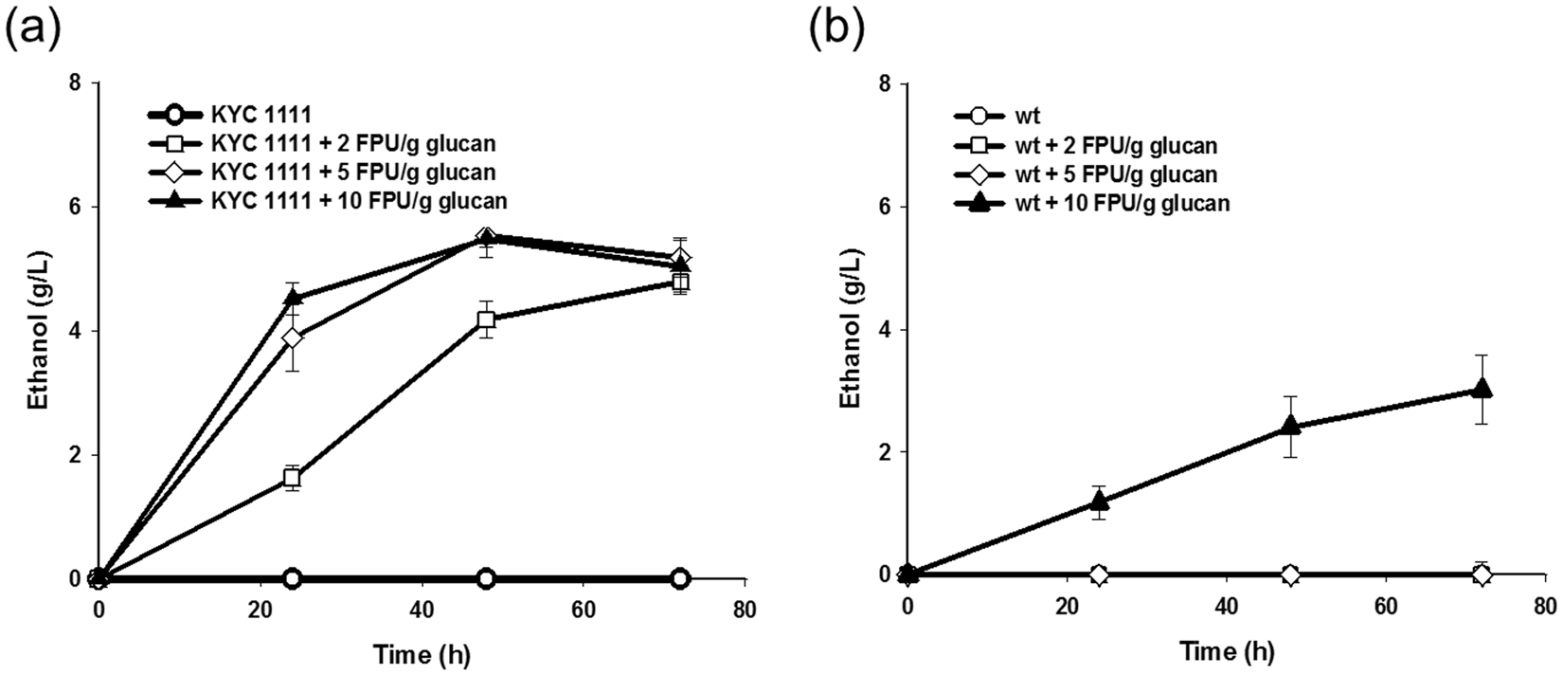
Ethanol production by the KYC 1111 mixture. The recombinant mixture KYC1111 was fermented using 2% MI-Avicel with several concentrations of Celluclast 1.5 L (0, 2, 5, and 10 FPU/g glucan). The graphs show the time course release of ethanol produced by the KYC1111 (**a**) and wild-type cells with empty vector (**b**) with no enzyme (circles), 2 FPU/g glucan (squares), 5 FPU/g glucan (diamonds), and 10 FPU/g glucan (triangles). Experiments were performed in triplicate and repeated three times.

For the production of ethanol from MI-Avicel, the cell mixture of 4 yeasts pre-cultured for 36 h, designated as the KRIBB yeast cocktail (KYC) 1111 for 1:1:1:1 mixture, was used as seed in the ratio of 30% (v/v) of the fermentation volume (final OD_600_ of 5.4 ± 0.6). However, no ethanol was produced (Fig. 6a), suggesting that the initial glucose concentration might have been too low (or almost absent). Under a fermentative condition, the rate of ethanol production is related to the available sugar concentration; thus, at a low initial concentration of glucose, most of the substrate is used for cellular maintenance rather than ethanol production^29, 30^. Therefore, to increase the initial concentration of glucose, a small amount of the commercial cellulase Celluclast® 1.5 L (2.85 mg/filter paper units (FPU)) was added to the medium before subculture. When Celluclast® 1.5 L was added to the KYC1111 at 2 FPU/g glucan, approximately 4.8 g/L of ethanol was produced from 20 g/L Avicel after 72-h fermentation (Fig. 6a). The percent yield (actual yield/theoretical yield) of ethanol was 48%. However, when the same amount of enzyme was added to the wild-type strain Y2805Δgal80, ethanol was not produced (Fig. 6b). This result indicated that the addition of 2 FPU of Celluclast® 1.5 L by itself did not affect the production of ethanol in the wild-type strain, but was sufficient to show a synergistic effect with the KYC1111 for the production of ethanol. When Celluclast® 1.5 L was added at an amount lower than 5 FPU/g glucan, the wild-type yeast strain did not produce any ethanol, whereas approximately 3 g/L of ethanol was produced with 10 FPU/g glucan of Celluclast® 1.5 L. By contrast, in the case of the KYC1111, ethanol production was enhanced with increasing amounts of Celluclast® 1.5 L, and the required amount of cellulase was saturated at 5 FPU/g glucan, resulting in an ethanol yield of 55% (Fig. 6a).

### Characterization of the mixture of the yeasts secreting cellulases

**Figure 7:**
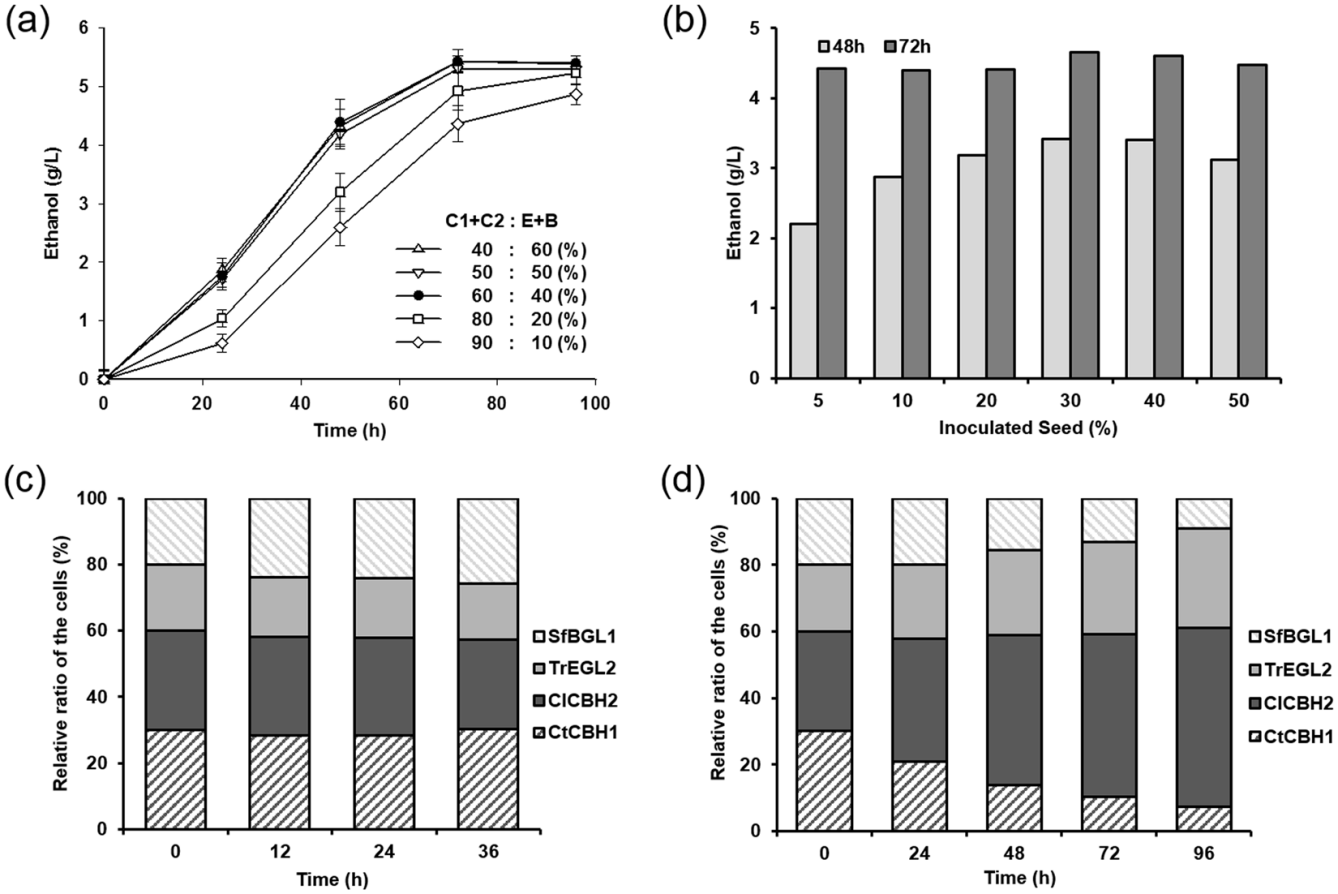
Optimization of the KYC composition. (**a**) Ethanol production from MI-Avicel by the KYC with various exocellulase compositions (from 40% to 90%) was analysed to find the optimal ratio of exocellulases in the KYC mixture. Experiments were performed in triplicate and repeated three times. (**b**) Ethanol production from 2% MI-Avicel was analysed at 72 h with various amount of seed (5–50%). (**c**) The relative ratio of the cells harbouring each cellulase expression vector was quantified by qPCR during the mixed cultivation of the KYC3322 either in YPD for 36h, and (**d**) in ethanol fermentation medium, including 2% (w/v) MI-Avicel, for 96h.

To determine the optimal ratio of the yeast mixture, the cells secreting CBH1, CBH2, EGL, and BGL were mixed at various ratios before the co-fermentation process. The ratios of exocellulases to EGL and BGL were varied at 4:6 (KYC2233), 5:5 (KYC1111), 6:4 (KYC3322), 8:2 (KYC4411), and 9:1 (KYC91). Although the difference in ethanol production among these ratios was not very large, ethanol production was higher when exocellulase accounted for 60% of the total cellular mixture (Fig. 7a), similar to the naturally secreted cellulase from cellulolytic fungi. Therefore, we used this ratio of exocellulases to other components for the subsequent experiments.

To determine the optimal amount of seed yeast for fermentation of the KYC, 5% to 50% (v/v) of inoculum was used. The recombinants, i.e., cells secreting each cellulase, were cultured separately in YPD medium for 36 h until the activity of each secreted cellulase reached its maximum, and the amount of cells was quantified by optical density analysis. Subsequently, the culture mixture at 5–50% (v/v) of the total reaction volume was inoculated in ethanol-fermenting medium at a ratio of 3:3:2:2 of CBH1, CBH2, EGL, and BGL (equivalent to a ratio of 6:4 for exocellulase to others). Ethanol production increased with the amount of seed until 30% at 48 h (Fig. 7b). Therefore, 30% seed was applied to the bioethanol fermentation at the next step.

Since we used a mixture of yeast strains secreting cellulases instead of a single cell in this process, the maintenance of the cellular ratio was analysed. To detect the relative ratio of the four cell types in the mixture during fermentation, the copy numbers of cellulase genes were determined by quantitative (q)PCR. When the cell mixture was cultured in YPD medium, the relative ratio of the cells was maintained (Fig. 7c). However, during ethanol fermentation using MI-Avicel as the substrate, the percentage of cells secreting CBH2 and EGL increased from 30% to 55% and from 20% to 30%, respectively (Fig. 7d). This means that the cellular ratio is affected by the carbon source, and a tailored cellular ratio was determined according to the changed fermentation conditions.

### Production of ethanol from rice straw with CBP

**Figure 8:**
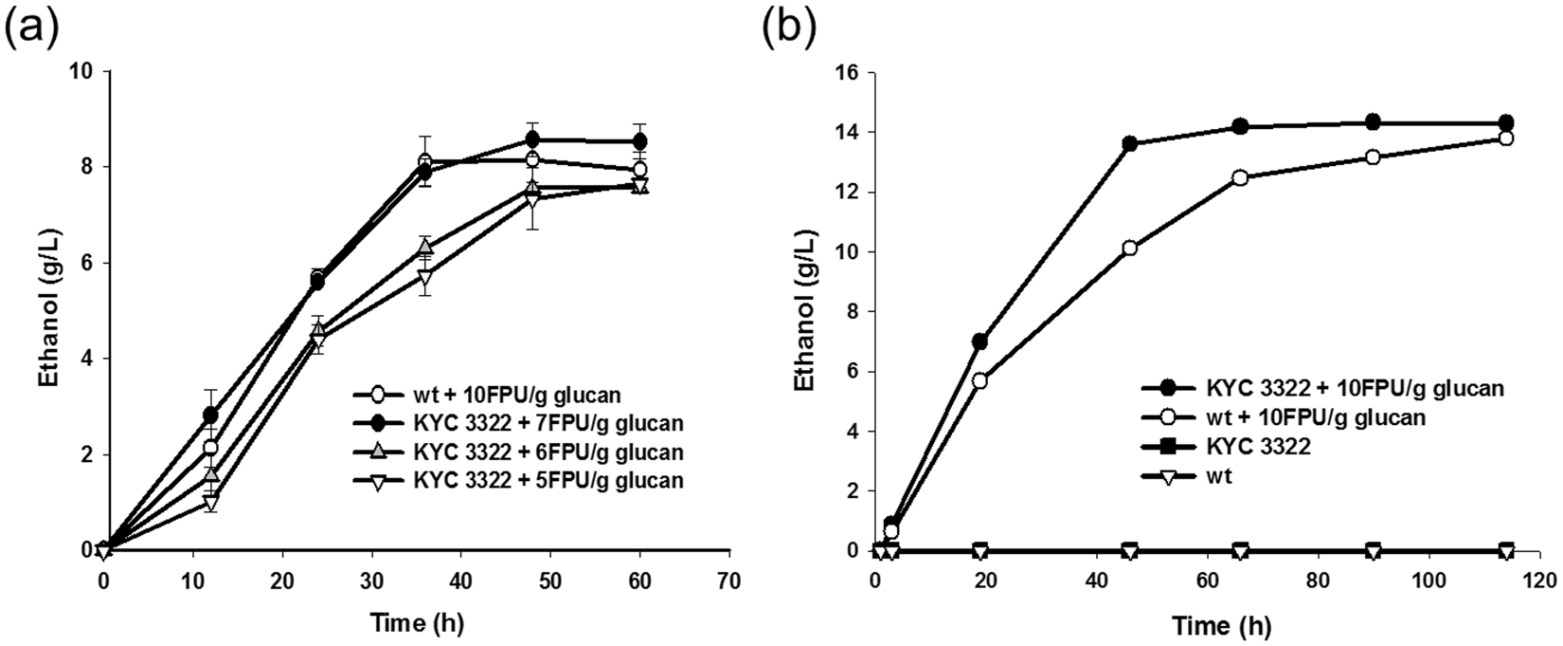
Ethanol fermentation from the cellulosic biomass using the KYC3322. (**a**) Ethanol production from pre-treated rice straw including 20 g glucan, using wild-type cells with 10 FPU Tec-mix/g glucan and the KYC3322 with 5–10 FPU Tec-mix/g glucan was compared in 100-mL flask culture. The values are the means of three repetitions of individual experiments ± standard deviations. (**b**) Ethanol production from pre-treated rice straw including 35 g glucan in 2-L fermentations by KYC3322 and wild-type yeast cells with 10 FPU Tec-mix/g glucan was analysed every 24 h for 5 days.

Given that commercial cellulase was still required for ethanol production in this KYC system, we next analysed the possible reduction in the amount of commercial cellulases required using this system. We compared ethanol production from 3% rice straw using wild-type cells with 10 FPU/g glucan of a mixture of Cellic® CTec2 and HTec2, with 70% CTec2 and 30% HTec2^31^ (hereafter referred as Tec-mix, 2.44 mg/FPU), and the KYC3322 with 5–10 FPU/g glucan of Tec-mix. In fermentation for bioethanol production using pre-treated rice straw, the ethanol productivity of wild-type cells with 10 FPU/g glucan of Tec-mix was equal to that of the KYC3322 with 7 FPU/g glucan of the extra enzyme of Tec-mix (Fig. 8a). As a result, the KYC3322 could replace approximately 3 FPU/g glucan of Tec-mix in the bioreactor. This approach could reduce the total production cost by 8–15% because the cost of the commercial enzyme accounts for approximately 25–50% of the total cost. The strain mixture KYC3322 expressing CtCBH1, ClCBH2, TrEGL2, and SfBGL1, accounting for 30% of the total volume, was inoculated into the ethanol fermentation medium with 10 FPU Tec-mix/g glucan, which resulted in approximately 14 g/L of ethanol production (percent yield of 78.7%) from the NaOH pre-treated rice straw containing 35 g/L glucan after 46 h with a productivity of approximately 0.30 g·L^−1^·h^−1^. However, the wild-type strain produced approximately 13.5 g/L of ethanol (percent yield of 77.1%) after 116 h with productivity of approximately 0.11 g·L^−1^·h^−1^. Consequently, co-fermentation with recombinant yeasts secreting different cellulases resulted in approximately 3-fold higher productivity than the wild-type yeast cell (Fig. 8b).

## Discussion

High-level secretion of functional cellulases is required for the economic production of bioenergy and biochemicals from lignocellulosic biomasses by non-cellulolytic organisms such as *S. cerevisiae* ^32^. However, it is difficult to express and secrete cellulases in a sufficient amount to effectively degrade crystal celluloses^13, 33^. Several studies have focused on the secretion of cellulases from yeast, a non-cellulolytic ethanol fermentation microorganism. Recently, the secretion of EGL and BGL using various signal peptides was comprehensively analysed^4, 34^, and Ilmen *et al*.^12^ identified exocellulases that are highly expressed in yeast. For secretion of the target enzymes, MFα or the native signal sequence is generally used^12, 20, 35^. However, because there is no omnipotent signal peptide for all genes, we screened optimal fusion partners for 7 CBH1genes (Cel7A), 3 CBH2 genes (Cel6A), EGL and BGL originating from various microorganisms using the recently described TFP system.

With the help of the TFP system, we identified good fusion partners for enhanced secretion of cellulases. The dominant candidates for secretion include: TFP 13 for HgCBH1, NfCBH1, TrCBH1, TrCBH2, CfCex, PaCel1, PaCel2, and ClCBH2; and TFP 19 for TrEGL2 and SfBGL1. TFP 13 contains 174 amino acids (aa) from the N-terminal of Hsp150 with an 18-aa signal peptide. Previously, a 321-aa fragment from the N-terminal of Hsp150, designated as Hsp150Δ, was used for the secretion of heterologous proteins in yeast^36^. Hsp150Δ consists of a signal sequence (18 aa), subunit I (54 aa, facilitating translocation into the endoplasmic reticulum lumen), and subunit II (composed of 11 repeats of a homologous, mostly 19 aa, proper folding fusion partner)^37^. However, in this study, we were able to induce the secretion of CBHs using only 174 aa of Hsp150, indicating that this sequence can serve as a useful secretion fusion partner for other proteins. TFP 19 contains 124 aa from the N-terminal of the soluble cell wall protein Scw4, which has glucosidase activity. The use of Scw4 as a fusion partner has not been reported until now. The target protein-specific function of TFP is still unknown but it may play an important role in proper folding of target proteins in the ER and/or trafficking to the Golgi complex.

The cellulolytic activity (Avicel conversion to glucose) of the most highly secreted CBHs, CtCBH1 and ClCBH2, in the cell-free culture supernatants was greater than 7% in 48 h, which is lower than that reported in a previous study (6–10% hydrolysis of Avicel)^12^. This difference may be related to the fact that Avicel PH-105 was used in the previous study, which has a smaller particle size than Avicel PH-101 used in the present study (~50 μm); this size difference might make it easier to be degraded by cellulases. In addition, we used high-performance liquid chromatography (HPLC) analysis instead of a 3,5-dinitrosalicylic acid (DNS) assay, used in the previous study, to measure the amount of glucose resulting from the enzymatic saccharification of crystalline cellulose. There are a few reports indicating the limitation of the DNS assay for the detection of reducing sugars from saccharified cellulose, which showed a higher value of glucose than that obtained with other analytical methods^38^. We used MI-Avicel to evaluate the activity of the cellulase mixture in the culture supernatant and the consequent ethanol production, and found a 50% increase in cellulose hydrolysis using MI-Avicel instead of Avicel, as previously reported^39^.

In this study, higher ethanol production from the MI-Avicel was achieved when exocellulases accounted for 60% of the total cellulases (KYC3322 culture mixture). However, there is no fixed exocellulase/endocellulase optimal ratio for cellulose hydrolysis. The hydrolysis is achieved by the synergistic action of EGLs, CBHs, and BGL. Most cellulolytic microorganisms produce an array of these enzymes, and the relative roles of the components are not easily defined^40^. It was reported that exocellulases accounted for approximately 70% of the total cellulases produced by *T. reesei* and other strains^41^. Experimental data have revealed high optimum exocellulase/endocellulase ratios in many cases; the optimum ratio of TrCBH2 and EGLs acting on various crystalline substrates was approximately 95:5, whereas the optimum ratio of TrCBH1 and EGLs acting on the same substrates was found to be approximately 1:1^42^. These findings suggest that the optimum ratio of cellulases may be dependent on the substrate used. Therefore, for maximal production of ethanol from various substrates, the optimum ratio of the enzymes might need to be determined on a case-by-case basis. In this respect, our co-fermentation of a cellular mixture has advantage. We used a mixture of cells secreting cellulases instead of a single cell expressing all cellulases because the optimal combination of cellulases is dependent on both the pre-treatment condition and the biomass source^32^, and many auxiliary proteins are needed for efficient cellulose degradation^33^. To analyse the maintenance of the cellular ratio during fermentation, the number of yeast cells expressing each cellulase was calculated by qPCR based on the relative copy number of the cellulase expression plasmid in the yeast cells. Although the relative ratio of the cells was maintained in YPD medium, the ratio of the cells was changed when cultured in ethanol fermentation from MI-Avicel. At the start of the culture, cells secreting CBH1, CBH2, EGL, and BGL were mixed together at a 3:3:2:2 ratio. Over time, however, the proportion of CBH2 and EGL increased to 80% of the total transcripts on the crystalline cellulose. The reason for this increase is still unclear, but it could be suggested that the proportion of mixed culture can be changed for adaptation to the substrate condition. Further investigation will be required for optimization of the seed condition for co-fermentation using different recombinant yeasts.

When the cellulase mixture was used for production of ethanol from pre-treated rice straw, the saccharification process with 10 FPU Tec-mix alone and the mixture of cells secreting cellulases, KYC3322 with 7 FPU Tec-mix showed similar rates of ethanol production. The filter paper assay showed that the cellulase activities of the Tec-mix and KCC1111 were 0.41 FPU/mg and 0.13 FPU/mg, respectively. This suggests that the amount of commercial enzyme can be reduced by approximately one third simply by using a mixture of yeast strains secreting cellulases.

Most of the reported yeast CBP systems used a single cell for the expression of multiple cellulases^35^. To develop a cellulolytic yeast consuming cellulosic biomass as a raw material, a minimum of four cellulases (2 CBHs, an EGL, and a BGL) should be expressed and secreted or displayed on the surface of a yeast cell. For the simultaneous utilization of hemicellulose, several more genes encoding xylanase are required. Insertion of multiple gene expression cassettes to a single yeast is not easy, and engineered cells are often genetically unstable. In contrast, the one-by-one yeast system developed in this study can eliminate such difficulties, especially for a synergistic hydrolysing enzyme system such as cellulase. The system can be easily adaptable for other related cellulolytic enzymes. To maximize the secretion of different cellulases, we have applied the TFP system. Although the secretion level of cellulases was apparently improved, it is still far from perfect. We do not rule out the possibility of an enzyme imbalance during co-fermentation of yeasts secreting different cellulases due to the different growth rates among strains and/or the instability of 2 micron plasmid. Therefore, further monitoring of each cell behaviour during co-fermentation under different seed ratios and culture conditions will be required for the practical application of our system.

At present, although the KYC3322 can be used simply as a cellulase saver, we expect that it can be further improved to be used as an independent cellulase for biomass degradation by the engineering of cellulases, a cellulase secretion system, and the host strain. For further improvement of cellulose hydrolysis and ethanol production, we can attach CBM to the catalytic domain of CBH1 to improve the activity of cellulases as reported previously^12^. In addition, the proper folding of cellulases with many disulphide bonds (e.g., 26 cysteines for CtCBH1 and 10 for ClCBH2, which makes it difficult to achieve the correct structural conformation) can be enabled by overexpression of single endoplasmic reticulum-resident foldases or chaperones^43^. Moreover, cellulase secretion can be increased by identification of novel fusion partners and the addition of helper proteins such as GH61 or CBM33^44, 45^, and engineering of secretory pathways in *S. cerevisiae* ^11^ would be used for improvement of a cellulolytic ethanol production system.

To reduce the production cost of bioethanol, many integrated processes such as SSF, SSCF, and CBP have been proposed. CBP is a perfect system for the production of ethanol, but a system for cellulase production and xylose-glucose co-utilization has not yet been well established. Therefore, we improved the secretion level of each cellulase and achieved the one-pot fermentation of recombinant yeasts secreting different cellulases. This is the first report of the consolidated bioprocessing for the production of bioethanol from rice straw by one-pot fermentation of recombinant *S. cerevisiae* strains secreting different cellulases. Finally, to construct a successful CBP strain for the production of cellulosic ethanol, we will use an expanded mixture of cells as a more effective and convenient system, which would require mixing yeast strains secreting hemicellulases such as β-xylanase and β-xylosidase. In this study, we used a mixture of the cells that secrete cellulases as ethanol producers. Such a mixture can be applicable to the design of sugar platforms in biorefineries to break down biomass into sugar monomers for fermentation or other biological processing.

## Methods

### Strains, media, and enzymes

*Escherichia coli* DH5α [F^-^ *lac*ZΔM15 *hsd*R17(r- m-) *gyr*A36] was used for the general recombinant DNA techniques. The haploid yeast strains *S. cerevisiae* Y2805 (*Mat* α *pep4*::*HIS3 prb1 can1 his3–200 ura3–52*) and Y2805Δ*gal80* (Y2805 *gal80*::*Tc190*) were used as hosts for the expression of cellulases^26^. All yeast transformations were performed using the lithium acetate method^46^. Transformants harbouring cellulase expression plasmids were selected on plates with a synthetic defined medium lacking uracil (SD-ura; 0.67% yeast nitrogen base without amino acids, 0.077% -ura dropout supplement, 2% glucose, pH 5.6–6.0). Yeast cells were generally grown on YPD (1% yeast extract, 2% bacto peptone, and 2% glucose) medium supplemented with the necessary compounds as required at 30 °C. The commercial cellulases Celluclast® 1.5 L, Cellic® CTec2, and HTec2 were purchased from Novozymes (Bagsvaerd, Denmark); Novozyme A188 was from Sigma-Aldrich Co. (St. Louis, MO, USA). The protein concentration was measured by the Pierce™ BCA Protein Assay Kit (Thermo Fisher Scientific, Waltham, MA, USA), and bovine serum albumin was used as the standard.

### Screening of cellulase-secreting yeasts using the TFP system

Information on the cellulase genes introduced in this study is summarized in Table 1, and the primer sets used are described in Supplementary Table S1. All the cellulase genes optimized based on *S. cerevisiae* codon usage were synthesised by Bioneer (Daejeon, Korea) except for the CfCex gene, which was amplified by PCR from the genomic DNA of *Cellulomonas fimi*. To express the *CBH1*, *CBH2*, *EGL*, and *BGL* genes using the TFP system^26^, the mature regions of the respective genes were amplified with forward and reverse primer sets (Supplementary Table S1). The cellulase expression vectors were directly constructed by *in vivo* recombination between the *Swa*I-digested TFP vectors and target genes (Fig. 2a). For secretion of the cellulases with their own signal sequences, amplified cellulase genes containing the signal sequence were integrated into the *Swa*I-digested YGaSW vector by *in vivo* recombination (Fig. 2b). To analyse the secreted proteins, recombinant *S. cerevisiae* Y2805 Δ*gal80* harbouring a cellulase expression vector was cultivated in a 20-mL test tube containing 3 mL broth medium for 40 h. Then, 0.6 mL of the culture supernatant was mixed with 0.4 mL of cold acetone. After 2-h incubation at −20 °C, the proteins were precipitated by centrifugation for 15 min at 10,000 × *g*. The pellets were freeze-dried, resuspended in 1 × SDS-PAGE sample buffer (Bio-Rad, Hercules, CA, USA), and analysed on 12% Tris-glycine gels under denaturing conditions by staining with Coomassie blue.

### Fed-batch fermentation of yeast strains

To prepare the seed culture, the yeast strains Y2805Δgal80/NS-CtCBH1, Y2805Δgal80/TFP13-ClCBH2, Y2805Δgal80/TFP19-TrEGL2, and Y2805Δgal80/TFP19-SfBGL1 were inoculated from the stock plate into SD-ura medium (50 mL) on a rotary shaker at 180 rpm and 30 °C for 24 h, respectively. After the first pre-culture, the cells were inoculated into the second pre-culture medium (200 mL of YPD) at 180 rpm, 30 °C for 24 h, and then inoculated into the fermenter containing 2 L of YPD medium. The fed-batch fermentation was carried out in a 5-L capacity of Jar fermenter (Fermentec, Cheongju, Korea) at 30 °C for 48 h. A feeding medium containing 300 g glucose and 150 g yeast extract (per liter) was used after depletion of glucose. The hourly feeding rate was manually increased from 2 to 10 g/L of carbon source based on cell growth. The fermentation conditions were set at an agitation rate from 300 rpm to 900 rpm, a pH controlled with NH_4_OH at 5.5, and an air-flow rate between 1.0 and 2.0 vvm. The concentration of total secreted protein (g/L) of each strain was quantitated by BCA assay using a 48-hour sample after fed-batch fermentation. Then, the secreted cellulase was quantified indirectly by determining band intensity on SDS-PAGE using ImageJ software.

### Enzyme activity assay

The enzyme activity assay was performed as described previously with some modification^12^. Secreted CBH activity was determined by measuring the quantity of glucose released from hydrolysed-insoluble crystalline cellulose, Avicel® PH-101 (Sigma Aldrich Co.). The yeast culture supernatant (600 μL) was mixed well to yield 600 μL of a solution containing 2% substrate and 0.04% sodium azide, and 0.5% (v/v) Novozyme A188 in 50 mM acetate buffer (pH 5.0) at 1,100 rpm, 50 °C. The glucose released at various reaction times, 0 h, 24 h, and 48 h, was detected using HPLC with an Aminex HPX-87H column (Bio-Rad). The HPLC analysis was performed at a flow rate of 0.6 mL/min with 5 mM sulphuric acid as the eluent at a column temperature of 65 °C by refractive index detection. Activity was expressed as the percentage of Avicel hydrolysed as described previously^12^. For analysis of cellulose hydrolysis by the endocellulase secreted from yeast, 50 μL of the supernatants were incubated with 150 μL of 50 mM sodium citrate buffer (pH 4.8) containing 1% CMC at 50 °C for 10 min. The development of colour in the solution mixture was then measured at 540 nm after boiling for 10 min with 700 μL DNS reagent and cooling. The reducing sugars released from CMC were analysed using a modified DNS assay. One unit of EGL activity was defined as the amount of the enzyme hydrolysing 1 μmol of reducing sugars per minute. The specific activity of BGL was measured as described previously with minor modifications^47^. To measure the activity of BGL enzyme, 100 μL of the culture supernatants were incubated for 15 min at 50 °C with 1 mM *para*-nitrophenyl-β-d-glycopyranoside (*p*-NPG) in 100 mM citrate phosphate buffer (pH 5.0). After addition of 30% Na_2_CO_3_ to stop the reaction, the amount of *p*-nitrophenol (*p*-NP) released from the reaction was detected at 410 nm. One unit of BGL activity was defined as the amount of enzyme required to liberate 1 μmol of *p*-NP per minute. To analyse the cellulolytic activity of the cellulase mixture, the glucose released by the cellulase mixture was detected using HPLC as described above. All experiments were performed in triplicate. The cellulase activity of the mixed culture supernatant was analysed by the NREL filter paper assay^48^ and reported in FPU/mg.

### Pre-treatment of cellulosic materials

Pre-treatment against 2% Avicel was conducted with MI at 25 °C for 5 min with thorough mixing, and then washed eight times with distilled water as described previously^39^. Rice straw and giant silver grass were obtained from a province in Korea, and palm EFBs were provided by a local palm oil processing company in Malaysia. For pre-treatment of cellulosic biomass, 400 g (dry weight) of rice straw or giant silver grass were cut into 2-cm pieces and pre-treated using NaOH (2% final concentration) at 160 °C for 1 h at 150 rpm followed by overnight cooling to 25 °C. This was followed by washing with deionized water until the pH was neutralised, and then the material was dried at 60 °C until a constant dry weight was obtained. The pre-treated rice straws were milled to pass through a 2-mm screen^49^. EFBs, as another cellulosic substrate, were prepared as follows. In the first step, the EFBs were treated with 0.7% sulphuric acid (solid to liquid ratio of 1:9) at 130 °C for 30 min, and the second stage was treatment with 2% sodium hydroxide at 150 °C for 1 h. The washing step for treated EFBs was the same as that described above. The glucan content of the pretreated rice straw or EFB was analysed according to the standard procedures of the National Renewable Energy Laboratory (NREL, USA)^50^. Approximately 0.3 mg of the pretreated biomass was incubated with 3 mL of 72% sulfuric acid in 20-mL glass vial at 30 °C for 2 h at 200 rpm. After completion of the hydrolysis, the reaction solution was diluted to 4%. Then the sealed solution was heated in an autoclave reactor for 1 h at 121 °C. The autoclaved hydrolysate was cooled slowly at room temperature and neutralized with calcium carbonate. The supernatant of the neutralized solution was detected using HPLC with an Aminex HPX-87H column (Bio-Rad). The conditions for HPLC analysis were the same as those for enzyme activity analysis.

### Measurement of cell ratio during the mixed culture of yeast by qPCR

For mixed-cell cultivation in YPD media for qPCR, each cell type secreting cellulase was grown in 250-mL baffled flasks containing 50 mL SD-ura medium at 180 rpm for 24 h. The KYC (KRIBB yeast cocktail) was made from the cells cultured in SD-ura media by inoculation at a CtCBH1:ClCBH2:TrEGL2:SfBGL1 ratio of 3:3:2:2, and grown at 30 °C in 50 mL of YPD broth for 36 h with sampling at 12-h intervals. To prepare samples during ethanol fermentation by KYC for qPCR, the samples were collected every 24 h until 96 h. Total DNA was extracted from the yeast cells and used as templates in qPCR. All primer sets from the respective cellulase sequences were optimized according to general primer selection conditions (product size 100–150; primer and product melting temperature 58–60 °C; primer GC content 50–60%) using the Primer3web program (http://primer3.ut.ee/). The qPCR was run on a QuantStudio™ 3 Real-Time PCR instrument (Applied Biosystems, Foster City, CA, USA). Standard curves for the four cellulase genes were generated from the serial-diluted plasmids using SYBR® Green Real-Time PCR Master mix (Applied Biosystems). The reaction was performed under the following condition: 40 cycles of denaturing at 95 °C for 20 s, annealing at 60 °C for 40 s, and elongation at 72 °C for 30 s. QuantStudio® Analysis Software (Applied Biosystems) was used to analyse the qPCR results such as efficiency of each primer set, cycle threshold values of the test samples, slope values, and melting curves. All samples were tested in triplicate.

### Ethanol production from biomass by *S. cerevisiae* in flasks

To analyse ethanol production from microcrystalline cellulose by the yeast cocktails secreting each cellulase, the KYC consisting of the four strains secreting each cellulase was used to ferment ethanol from 2% (w/v) MI-Avicel or 3% (w/v) pre-treated rice straw. Strains of the KYC were separately cultivated in YPD media until the optical density at 600 nm reached 18 ± 2; 7.5 mL of each culture solution was inoculated into 100 mL of ethanol fermentation medium (0.5% yeast extract, 0.5% peptone, 0.5% potassium phosphate, 0.2% ammonium sulphate, 0.04% magnesium sulphate), for a total 30% (v/v) KYC culture solution in final volume. The strains were then cultured for 96 h at 30 °C under similar anaerobic conditions at 100 rpm in the presence of the commercial cellulases, Celluclast or Tec-mix, with different concentrations (0, 2, 5, 10 FPU/g glucan). Accumulation of ethanol in the medium was detected using HPLC as described above.

### Consolidated bioprocessing using pre-treated rice straw in a bioreactor

The CBP was performed with a final working volume of 2 L ethanol fermentation medium, which contained 5% (w/v) pre-treated rice straw as a carbon source in a 5-L stirring batch bioreactor (Fermentec). After the KYC seeds were initially grown in SD-ura medium (100 mL) at 180 rpm, at 30 °C for 24 h, they were inoculated into YPD medium (500 mL) and grown with shaking at 180 rpm at 30 °C for 48 h. This was utilized as the final 30% (v/v) seed during ethanol fermentation. Ethanol was produced from KYC with Tec-mix added in a bioreactor with shaking at 100 rpm until 116 h. The analytical method to detect ethanol derived from rice straw was the same as the HPLC analysis used to detect glucose released from the substrate in the enzyme assay.

## Electronic supplementary material

Supplementary Information
