# Supplementary material for: Co-fermentation using Recombinant Saccharomyces cerevisiae Yeast Strains Hyper-secreting Different Cellulases for the Production of Cellulosic Bioethanol
Source: Sci Rep. 2017 Jun 30;7:4428. doi: 10.1038/s41598-017-04815-1 (PMC5493647; doi:10.1038/s41598-017-04815-1)
Supplement: Supplementary file 1 — Supplementary Information [file 41598_2017_4815_MOESM1_ESM.docx]

**Co-fermentation using Recombinant *Saccharomyces cerevisiae* Yeast Strains Hyper-secreting Different Cellulases for the Production of Cellulosic Bioethanol**

Cho-Ryong Lee ^1,2,*^, Bong Hyun Sung^1,2,*^, Kwang-Mook Lim^1^, Mi-Jin Kim^1^, Min Jeong Sohn^1^, Jung-Hoon Bae^1^, and Jung-Hoon Sohn^1,2^

*^1^Cell Factory Research Center, Korea Research Institute of Bioscience and Biotechnology (KRIBB), Daejeon 34141, Republic of Korea*

*^2^Department of Biosystems and Bioengineering, KRIBB School of Biotechnology, Korea University of Science and Technology (UST), Daejeon 34113, Republic of Korea*

**Supplementary Information**

Table S1. Primers used in this study for plasmid construction

Figure S1. Secretion of ClCBH2 by 24 TFP vectors.

Figure S2. Cellulose hydrolysis activity of secreted CBHs according to the culture time.

Figure S3. SDS-PAGE analysis for the secreted cellulases from recombinant yeast during fed-batch fermentation.

**Table S1.** **Primers used in this study for plasmid construction**

| Primer^a^ | Sequence (5’ – 3’) |
| --- | --- |
| ClCBH2-NS-F | GAATTTTTGAAAATTCAAGAATTCATGGCCAAAAAGTTGTTCATTACC |
| ClCBH2-F | GGCCGCCTCGGCCTCTGCTGGCCTCGCCTTAGATAAAAGAGCACCAGTAATAGAAGAAAGAC |
| ClCBH2-R | GTCATTATTAAATATATATATATATATATTGTCACTCCGTTCAAGTCGACTTAGAATGGTGGATTTGCG |
| CtCBH1-ss-F | GAATTTTTGAAAATTCAAGAATTCATGATGTATAAGAAGTTTGC |
| CtCBH1-F | GGCCGCCTCGGCCTCTGCTGGCCTCGCCTTAGATAAAAGACAACAAGCGTGTTCCCTC |
| CtCBH1-R | GTCATTATTAAATATATATATATATATATTGTCACTCCGTTCAAGTCGACTTACAGACACTGAGAATACC |
| HgCBH1-NS-F | GAATTTTTGAAAATTCAAGAATTCATGCGTACCGCCAAGTTTGC |
| HgCBH1-F | GGCCGCCTCGGCCTCTGCTGGCCTCGCCTTAGATAAAAGACAGCAAGCGTGTAGTCTCACC |
| HgCBH1-R | GTCATTATTAAATATATATATATATATATTGTCACTCCGTTCAAGTCGACTTACAAACATTGAGAGTACC |
| NfCBH1-F | GGCCGCCTCGGCCTCTGCTGGCCTCGCCTTAGATAAAAGACAACAGGTCGGTACTTCC |
| NfCBH1-R | GTCATTATTAAATATATATATATATATATTGTCACTCCGTTCAAGTCGACTTACAGGCATTGAGAGTAAAAG |
| TeCBH1-NS-F | GAATTTTTGAAAATTCAAGAATTCATGCTTAGAAGAGCTCTTTTGC |
| TeCBH1-F | GGCCGCCTCGGCCTCTGCTGGCCTCGCCTTAGATAAAAGACAACAAGCAGGCACTGCAACG |
| TeCBH1-R | GTCATTATTAAATATATATATATATATATTGTCACTCCGTTCAAGTCGACTTACGAAGCGGTAAAGGTCG |
| TrCBH1-F | GGCCGCCTCGGCCTCTGCTGGCCTCGCCTTAGATAAAAGACAGTCGGCCTGCACTCTC |
| TrCBH1-R | GTCATTATTAAATATATATATATATATATTGTCACTCCGTTCAAGTCGACTTACAGGCACTGAGAGTAG |
| TrCBH2-F | GGCCGCCTCGGCCTCTGCTGGCCTCGCCTTAGATAAAAGACAAGCTTGCTCAAGCGTCTG |
| TrCBH2-R | GTCATTATTAAATATATATATATATATATTGTCACTCCGTTCAAGTCGACTTACAGGAACGATGGGTTTG |
| PaCel1-F | GGCCGCCTCGGCCTCTGCTGGCCTCGCCTTAGATAAAAGACAGCAAGTGGGCACGCTC |
| PaCel1-R | GTCATTATTAAATATATATATATATATATTGTCACTCCGTTCAAGTCGACTTAGGAGGTGAAGGTGGAG |
| PaCel2-F | GGCCGCCTCGGCCTCTGCTGGCCTCGCCTTAGATAAAAGACAGGCTCCCGTGTACGG |
| PaCel2-R | GTCATTATTAAATATATATATATATATATTGTCACTCCGTTCAAGTCGACTTACAGCGGCGGGTTCG |
| CfCex-F | GGCCGCCTCGGCCTCTGCTGGCCTCGCCTTAGATAAAAGAGCGACCACGCTCAAGGAG |
| CfCex-R | GTCATTATTAAATATATATATATATATATTGTCACTCCGTTCAAGTCGACTCAGCCGACCGTGCAGG |
| SfBGL1-NS-F | ATTTTTGAAAATTCAAGAATTCATGTTGATGATAGTACAGCTTTTGG |
| SfBGL1-F | GGCCGCCTCGGCCTCTGCTGGCCTCGCCTTAGATAAAAGAGTCCCAATTCAAAACTATACC |
| SfBGL1-R | GTCATTATTAAATATATATATATATATATTGTCACTCCGTTCAAGTCGACTTAAATAGTAAACAGGACAG |
| TrEGL2-NS-F | ATTTTTGAAAATTCAAGAATTCATGAACAAGTCCGTGGCTCC |
| TrEGL2-F | GGCCGCCTCGGCCTCTGCTGGCCTCGCCTTAGATAAAAGACAGCAGACTGTCTGGGG |
| TrEGL2-R | GTCATTATTAAATATATATATATATATATTGTCACTCCGTTCAAGTCGACCTACTTTCTTGCGAGACACG |

^a^F: forward primer for TFP system, NS-F: forward primer of native signal sequence for protein secretion, R: reverse primer

**
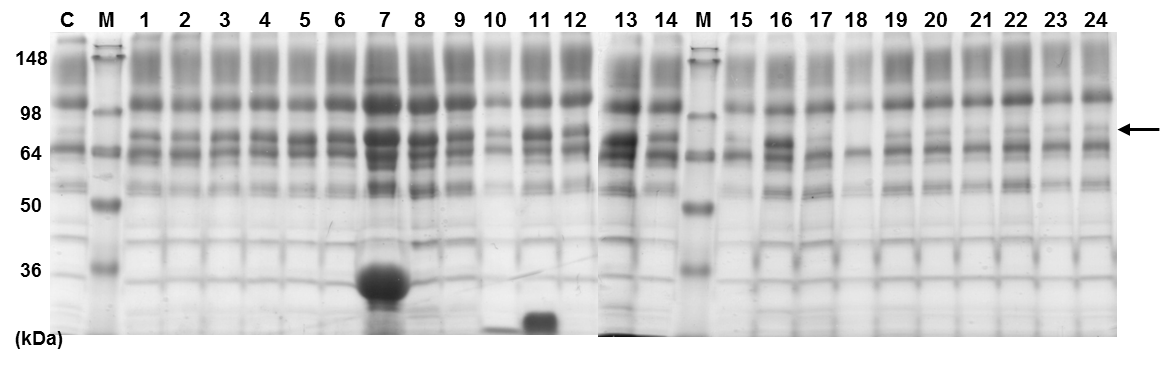
**

**Figure S1. Secretion of ClCBH2 by 24 TFP vectors.** SDS-PAGE analysis of ClCBH2 expressed by 24 selected TFPs. A 0.6-mL aliquot of the culture supernatant was analyzed on a 12% Tri-glycine gel after precipitation with acetone. M: standard protein size marker, C: host strain carrying a mock vector, lanes 1–24: each TFP (Table 2). The arrow indicates ClCBH2 secreted to the culture supernatant.

**
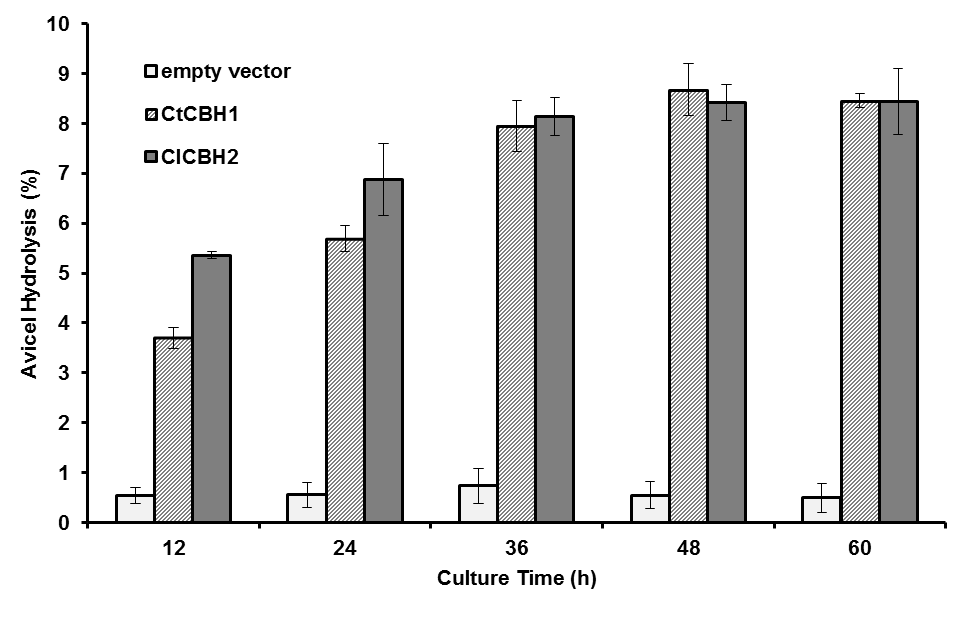
Figure S2. Cellulose hydrolysis activity of secreted CBHs according to the culture time.** The activity of Avicel hydrolysis was detected based on the amount of glucose (g/L) released after 36 h by secreted CBHs up to 60 h with 0.5% (v/v) Novozyme A188.


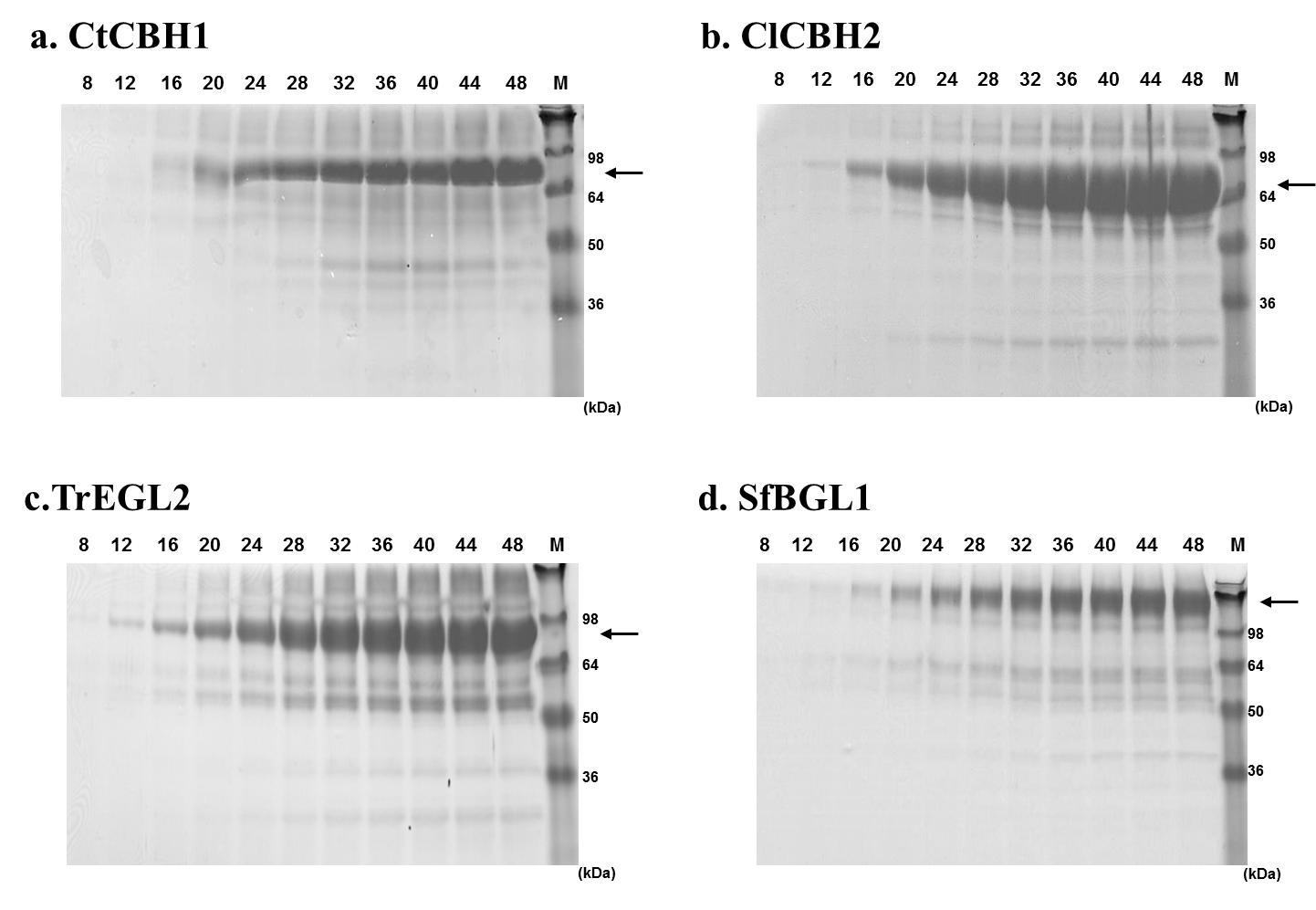


**Figure S3. SDS-PAGE analysis for the secreted cellulases from recombinant yeast during fed-batch fermentation.** Ten microliters of the culture supernatant was analyzed on a 12% Tri-glycine gel at a specific time of fermentation. M: standard protein size marker, lanes 8–48: supernatant of fermentation with 4-h time intervals during the 48-h culture.
